# Remediating reduced memory specificity in bipolar disorder: A case study using a Computerized Memory Specificity Training

**DOI:** 10.1002/brb3.1468

**Published:** 2019-11-20

**Authors:** Kris Martens, Keisuke Takano, Tom J. Barry, Emily A. Holmes, Sabine Wyckaert, Filip Raes

**Affiliations:** ^1^ Faculty of Psychology and Educational Sciences KU Leuven Leuven Belgium; ^2^ Ludwig‐Maximilians‐University of Munich Munich Germany; ^3^ Department of Psychology The University of Hong Kong Hong Kong Hong Kong; ^4^ Department of Psychology The Institute of Psychiatry King's College London London UK; ^5^ Department of Psychology Uppsala University Uppsala Sweden; ^6^ University Psychiatric Center KU Leuven, Campus Kortenberg Kortenberg Belgium

**Keywords:** autobiographical memory, bipolar disorder, Memory Specificity Training, reduced autobiographical memory specificity

## Abstract

**Objectives:**

Reduced autobiographical memory specificity (rAMS) is a vulnerability factor found across unipolar depression (UD), posttraumatic stress disorder (PTSD), eating disorder, schizophrenia, and bipolar disorder (BD). A group delivered psychological therapy training called Memory Specificity Training (MeST) remediates rAMS in UD and PTSD, with additional downstream effects on related psychological processes and symptoms. Its impact in BD is unknown. In this case study, we examined the impact of a computerized version of MeST (c‐MeST) on improving AMS and related symptoms and processes in participant with rapid cycling type I BD.

**Method:**

An experimental case study with an ABA design was used. During baseline (14 days, Phase A), the training phase (nine sessions across 17 days, Phase B), and a 1‐month follow‐up (Phase A), memory specificity, depressive symptoms, and related processes and symptoms were repeatedly measured.

**Results:**

Memory specificity increased significantly after the participant completed c‐MeST. Session‐to‐session scores indicated that AMS improved most from the in‐person baseline assessment to the first online session. All other measures of processes and symptoms deteriorated during the training phase but regressed to baseline during follow‐up.

**Conclusion:**

Memory specificity was improved as indicated by increased AMS from pre‐intervention measurement to 1‐month follow‐up. Other improvements in symptoms were not observed. Rather, some related maladaptive psychological processes and symptoms worsened during the training phase and regressed to baseline during follow‐up.

## Graphical abstract

In an experimental single case study autobiographical memory specificity was successfully remediated, from pre‐intervention measurement to 1‐month follow‐up, by using an online memory specificity training, in one person with rapid cycling Bipolar Disorder type I. Other improvements in symptoms were not observed. Rather, some related maladaptive psychological processes and symptoms worsened during the training phase and regressed to baseline during follow up.

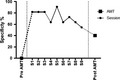

## 1 INTRODUCTION

Several cognitive vulnerabilities have been found in individuals with bipolar disorder (BD), including reduced autobiographical memory specificity (rAMS) (Scott, Stanton, Garland, & Ferrier, 2000). Despite the potential importance of rAMS within BD, and the existence of interventions that target rAMS (Barry, Sze, & Raes, 2019), no study has yet examined whether these interventions are effective at improving rAMS among people with BD, or whether doing so can be beneficial. In this experimental case study, we explored the modifiability of rAMS for a participant diagnosed with rapid cycling type I bipolar disorder (American Psychiatric Association, 2013) with a computerized version of Memory Specificity Training (MeST) (Raes, Williams, & Hermans, 2009), called c‐MeST (Takano, Moriya, & Raes, 2017). We do not intend to present this as a case study examining MeST as a stand‐alone treatment for BD but as an exploration of MeST's potential as an add‐on intervention as part of wider care management.

rAMS—or overgeneral autobiographical memory (OGM)—can be described as a difficulty retrieving specific, personal memories of events lasting less than a day. For example, when asked to retrieve a specific memory given the cue word “Happy,” the response “I remember I was happy when I first kissed my partner” refers to a specific autobiographical memory. However, people with rAMS may respond to this cue word with a categorical event that took place repeatedly (e.g., “I'm always happy seeing friends”) or an extended memory of an event lasting longer than 1 day (e.g., “I was happy when I was on holiday in France”).

rAMS is considered an enduring trait in unipolar depression (Farina, Barry, Damme, Hie, & Raes, 2018; van Vreeswijk & de Wilde, 2004) and is also found in posttraumatic stress disorder (PTSD) (Ono, Devilly, & Shum, 2016), eating disorders (Dalgleish et al., 2003), and in schizophrenia (Barry, Del Rey, & Ricarte, 2018). The presence of rAMS has also been examined in BD but evidence is more mixed. Compared to healthy controls (HC), people in remission from BD (type I) reported worse rAMS (Oertel‐Knöchel et al., 2012; Scott et al., 2000). Compared to people in remission from UD, Mansell and Lam (2004) found worse rAMS in people in remission from BD (albeit for negative cues only). This difference was not found in two other examinations (Tzemou & Birchwood, 2007; Young & Bodurka, 2016), but in both studies people in remission from BD and UD showed worse memory specificity compared to HC.

Evidence such as this, and evidence of other autobiographical memory problems among people with psychological problems, has resulted in the emergence of the field of memory therapeutics, as illustrated by recent reviews of such interventions in mood, anxiety, and stress‐related disorders (Barry et al., 2019; Hitchcock, Werner‐Seidler, Blackwell, & Dalgleish, 2017). The authors of these reviews reported that no studies have yet examined the impact of a memory‐therapeutic intervention among people with BD. One intervention, Memory Specificity Training (MeST) (Raes, Williams, et al., 2009), is regarded as a promising method for improving memory specificity and remediating related symptoms and processes in UD (Eigenhuis, Seldenrijk, Schaik, Raes, & Oppen, 2017; Raes, Williams, et al., 2009; Werner‐Seidler et al., 2018), PTSD (Maxwell et al., 2015), and Schizophrenia (Blairy et al., 2008; Ricarte, Hernández‐Viadel, Latorre, & Ros, 2012). A recent meta‐analysis confirms these effects (Barry et al., 2019) and concluded that MeST has also been found to improve other processes associated with rAMS, such as deficits in problem solving, future‐oriented thinking, and hopelessness. The group training protocol of MeST was translated to a computerized application that can be offered individually: computerized MeST or “c‐MeST” (Takano, Moriya, et al., 2017). For this study, the same version of c‐MeST is used as in the examination of c‐MeST in healthy older adults with rAMS (Martens, Takano, et al., 2019) in which each session consists of 11 specificity exercises. The platform offers immediate feedback about the specificity of the answer, such that the participant can learn from feedback.

The principle aim of this study was to investigate whether c‐MeST increases memory specificity from pre‐intervention to 1‐month follow‐up for one participant with BD. A second research question concerned whether c‐MeST would impact depressive symptoms and associated processes. Conway and Pleydell‐Pearce (2000) proposed a model which states that autobiographical memories are retrieved by searching autobiographical knowledge which is hierarchically ordered from general, summarized information to event‐specific details. The CaR‐FA‐X model (Williams et al., 2007) describes the mechanisms which can disrupt the top‐down search through this hierarchy: Capture and Rumination (when conceptual self‐relevant information activates ruminative processes and thus capturing cognitive resources and disrupting the search down the hierarchy), Functional Avoidance (when the retrieval of specific memories is passively avoided to regulate negative affect), and impaired eXecutive Control (when deficits in executive resources limit the ability to conduct a successful retrieval search). rAMS is found to be associated with other forms as avoidance as well. For example, Hermans, Defranc, Raes, Williams, and Eelen (2005) found that, among people with UD, rAMS was associated with social avoidance, avoidance of thoughts about painful experiences or feelings, and suppression of negative thoughts. Tzemou and Birchwood (2007) found that people with UD and BD who had worse rAMS also reported fewer intrusive thoughts, perhaps due to a tendency to avoid these negative thoughts. These authors hypothesize that rAMS in BP reduces stress associated with life events, but also reduces awareness of emerging distressing affect, which might contribute to BP relapse. The authors suggest that CBT in BP should focus on counteracting a generalized avoidant style. This is exactly one of the aims for which MeST has been developed. Also, BD is associated with impaired problem solving (Scott et al., 2000; Tzemou & Birchwood, 2007) and future thinking (Boulanger, Lejeune, & Blairy, 2013).

Aspects of memory in BD might, however, also differ from UD. Barry and colleagues (Barry, Chiu, Raes, Ricarte, & Lau, 2018) describe in their review paper on the neurobiology of rAMS that although similar behavioral deficits were found in UD and BD, the neurophysiological underpinnings of AM recall between people with UD and BP differed. Memory retrieval in people with BD is characterized by memories with enhanced salience and emotional vividness and is processed as more self‐relevant compared to people with UD and HC (Oertel‐Knöchel et al., 2012; Young & Bodurka, 2016). Relatedly, it has been proposed that mental imagery (e.g., in vivid episodic recall) can act as an emotional amplifier in BD (Holmes et al., 2011). An emerging treatment approach is to target such mental imagery in BD to improve mood stability (Holmes et al., 2016). Recent guidelines for BD have called for innovations in psychological treatment, in particular interventions with a focus in on BD's mental imagery‐based memory (Goodwin et al., 2016).

In the current study, we investigate the impact of c‐MeST on memory specificity and on symptoms and processes related to the “Capture and Rumination” and “Functional Avoidance” domains of the Car‐FA‐X model. In particular, whether MeST improves a person's tendency to ruminate (CaR), worry (CaR), whether it reduces the presence and negative affect associated with unwanted thoughts and memories (CaR and FA), and whether it reduces the tendency to utilize thought suppression to regulate negative affect (FA). In addition, the impact of c‐MeST on future thinking is examined. A third research question regarded the experiences of the participant concerning the c‐MeST platform and the cue word exercises and feedback used within it.

## 2 METHOD

### 2.1 Participant selection

The recruitment setting was an university‐affiliated psychiatric hospital (UPC Kortenberg), where a psychiatrist (the fifth author) explained the study briefly to potential participants and distributed a leaflet with information about the study. Patients expressing interest were invited for a meeting with the first author in which they received additional information. General inclusion criteria for this study were (a) experiencing rAMS, operationalized as a score of less than 70% on the Autobiographical Memory Test (Williams & Broadbent, 1986), (b) being diagnosed with BD type I by the psychiatrist involved, (c) being stable or in remission and consulting a psychiatrist. The recruitment phase took place between May and August 2017. Our initial aim was to include at least three cases to comply to the evidence standards for an single case experimental design (Kratochwill et al., 2010), but unfortunately only one participant was included. In total, 12 patients with BD (seven women) were assessed, with a mean AMT score of 7.92 (*SD* = 2.78). Only two female patients with BD showed rAMS. One participant with BD type I (American Psychiatric Association, 2013) rapid cycling was willing to participate. The other patient showing rAMS (with an AMT score of 6/10) decided not to participate due to the perceived time cost of the study.

### 2.2 Participant description

The participant was a 40‐year‐old Belgian Caucasian woman with a diagnosis of BD type I rapid cycling. She had obtained a Masters‐level degree. She had her first episodes at age 27 and was diagnosed that year with BD type I rapid cycling. Since then, she has been unemployed and is receiving a disability allowance, based on the BD diagnosis, and has been doing volunteer work. The participant reported no history of any other diagnoses and mentioned no history of clear trauma, except for one traffic accident which did impact her condition. She had received different kinds of treatments, among which she had experienced two periods of day hospitalization. At the time of the study, she was seeing a psychiatrist and a clinical psychologist once every month. With regard to medication use, the participant was taking lithium (800 mg), plus auetiapine (600 mg), and aripiprazole (10 mg) at the time of the study. At the beginning of the study, the quetiapine was raised by 100 mg for a week, due to a hypomanic episode. Prior to participating in this study, the participant was discharged from an outpatient day treatment program. She lived alone.

### 2.3 The intervention

C‐MeST consists of nine sessions of eleven specificity exercises. In each specificity exercise, the participant was asked to retrieve a specific memory. Immediate feedback on the memory was provided. The participant completed each session on an online platform which contained instructions and tips about autobiographical memory specificity, similar to the instructions of the AMT but with more examples provided. Nine of the eleven specificity exercises consisted of cue words: three positive (e.g., safe), three negative (e.g., guilty), and three neutral (e.g., forest). Similar to the structure of therapist‐provided “off‐line” MeST, each of the sessions included one exercise about a memory of an event that occurred in the previous day and also for an event that occurred within the same day (without cue words, referred to as *exercises of the day*). The Web site used the computerized scoring algorithm for the Autobiographical Memory Test (Takano, Ueno, Moriya, & Mori, 2017) to score responses. If the entry was not specific enough, the participant was encouraged with automated feedback to re‐enter the memory (or another memory) but with more episodic details. For each cue word, the participant obtained three chances to enter a specific memory, otherwise the next exercise was automatically presented. If the participant succeeded in providing a specific memory, she was invited to provide more spatiotemporal and contextual details on the next page (i.e., “Where did it happen? When did it happen? How long did it take? Who else was there? What can you see, hear, smell or taste? What kind of day was it?”). The participant was able to skip a cue word if she wished to do so. There was no time limit per question.

### 2.4 Study design and procedure

During the meeting of the participant and the first author, after assessing memory specificity, c‐MeST was introduced to the patient. As the participant recognized the phenomenon of rAMS and expressed the wish to improve this skill, it was explained that by training this memory specificity might improve and potentially impact secondary processes as well. The participant received a personalized file with a schedule of which day she had to train and/or fill out questionnaires, based on a baseline length of 14 days. Each day when the participant was assumed to fill out a questionnaire or complete a training session, an invitation by e‐mail was sent automatically using the software Boomerang (http://www.boomeranggmail.com) to remind her.

**Figure 1:**
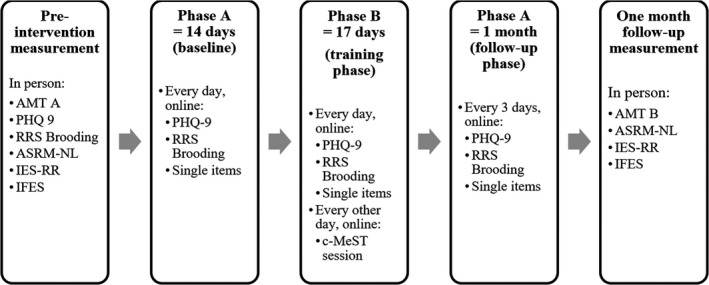
Illustration of the research design. AMT, Autobiographical Memory Test; ASRM‐NL, Altman Self‐Rating Mania Scale; c‐MeST, Computerized Memory Specificity Training; IES‐RR, Impact of Events Scale; IFES, Impact of Future Events Scale; PHQ 9, Patient Health Questionnaire 9; RRS Brooding, Ruminative Response Scale Brooding Subscale

Symptoms and related psychological processes were repeatedly measured throughout the three phases: a first measurement a baseline of 14 days (Phase A), a training phase of 17 days (Phase B) with a training session every other day, and a follow‐up phase of 1 month (Phase A). The participant was instructed to fill out the measures every day during Phase A and Phase B, and once every 3 days during follow‐up. In addition to the repeated measures, some processes were only measured at pre‐training and at the end of follow‐up: impact of events, impact of future events, and manic symptoms. The full study design can be found in Figure 1.

No blinding was used, although all repeated measures are generated without the presence of an experimenter. The participant did not receive any compensation. The study received institutional ethical approval of the Social and Societal Ethics Committee of the University of Leuven (G201412113).

### 2.5 Outcomes

1. **Autobiographical Memory Specificity** was measured using a verbal version of the Autobiographical Memory Test (Dalgleish et al., 2007; Williams & Broadbent, 1986).
2. **Depressive symptomatology** was measured using The Patient Health Questionnaire 9 (Kroenke, Spitzer, Williams, & Löwe, 2010) (Dutch translation).
3. **Rumination** was measured using the Ruminative Response Scale–Brooding subscale (RSS Brooding) (Raes, Schoofs, et al., 2009; Treynor, Gonzalez, & Nolen‐Hoeksema, 2003).
4. **Manic Symptoms** were measured using the Altman Self‐Rating Mania Scale (ASRM‐NL, Dutch translation). (Altman, Hedeker, Peterson, & Davis, 1997).
5. **Impact of Events** was assessed using the Impact of Event Scale–Revised (IES‐R, Dutch translation by TZP Psychotrauma 2006). (Weiss & Marmar, 1995).
6. **Impact of intrusive prospective imagery** was assessed using the Impact of Future Events Scale (IFES (Deeprose & Holmes, 2010), translated ad hoc to Dutch).
7. **Items for Repeated Measurements.** Eight single items were added to the online platform for repeated measures that capture process change. Items are shown in Figure 3.
8. **Measures of c‐MeST training experiences.** After each c‐MeST session, the participant was asked three closed and two open questions regarding feasibility.

A more extensive description of each measure can be found as Appendix S1 in the online publication.

### 2.6 Analysis

For the current study, four categories of measures are analyzed: memory specificity, related symptoms, related processes, and feasibility. The participant's responses within each c‐MeST session were scored manually by the first author after treatment completion. C‐MeST sessions were scored as the number of trials for which the participant's first answer was classified as a specific autobiographical memory, in accordance with the logic of the AMT, resulting in a maximum of 11 points per session. Inter‐rater reliability between automated scores of the classifier and manual post‐hoc scores were substantial with a Cohen's *κ* of 0.66, *p* < .001. As such, in addition to examining change in memory specificity from pre‐intervention to follow‐up, session‐to‐session change was also examined.

Regarding symptoms, we assessed manic symptoms at pre‐intervention and follow‐up, and depressive symptoms, happiness, and sadness as repeated measures using PHQ‐9 and two single items. Regarding related processes, rumination was measured repeatedly using RRS Brooding and a single item. Worrying was measured using a repeated single item. For visual analysis of all repeated measures, the software “Single Case Data Analysis” (Bulté & Onghena, 2013) was used, which uses the R packages SCRT, SCVA, and SCM. For visual analysis with SCVA, the level of each phase using mean scores and the trend of each phase using least squares regression are shown (Bulté & Onghena, 2011). The impact of events was assessed at pre‐intervention and follow‐up using the IES‐R. The IES‐R consists of three subscales from which we created three questions that were included during the repeated measures assessment: unwanted thoughts or images, thought suppression, and being tense when a painful memory arises. These were combined for visual analysis. The impact of future thinking was assessed only at pre‐intervention and at follow‐up by using the IFES. Regarding feasibility, the participant was offered questions after each session and the participant was asked to provide feedback at the follow‐up measurement.

### 2.7 Ethical approval

The study received institutional ethical approval of the Social and Societal Ethics Committee of the University of Leuven and all participants filled out and signed an informed consent form.

## 3 RESULTS

### 3.1 Compliance and feasibility

The participant completed all exercises and filled out all repeated measures throughout the three phases, resulting in sufficient data for visual analysis. Although she was instructed that filling out the repeated measures during 1‐month follow‐up once every 3 days would suffice, during the first half of follow‐up she continued to fill out the questionnaires each day.

Regarding feasibility (see Appendix S2 in the online publication), the participant experienced the cue words, on average, as appropriately difficult (*M* = 5.5, *SD* = 2.00) and reported that the feedback she received was typically correct (*M* = 8.75, *SD* = 0.89). The length of the sessions was, on average, experienced as between “just right” and “a bit too long” (*M* = 3.63, *SD* = 0.52). Recurrent themes in participant's open‐ended responses were that she experienced the training as interesting and challenging, and at the 1‐month follow‐up, she reported that MeST made her more aware of events happening throughout the day. She confirmed during the follow‐up assessment that sessions lasted a bit too long and the training (17 days, training every other day) lasted too long as well.

### 3.2 Effects of c‐MeST on memory specificity during training and at 1‐month follow‐up

The participant exhibited an increase in specificity from pre‐intervention (AMT A = 0 out of a possible 10 specific responses) to 1 month after intervention completion (AMT B = 4/10). According to our inclusion criterion, the participant's score at follow‐up is still in the range of what we would define as rAMS (<70% on the AMT).

**Figure 2:**
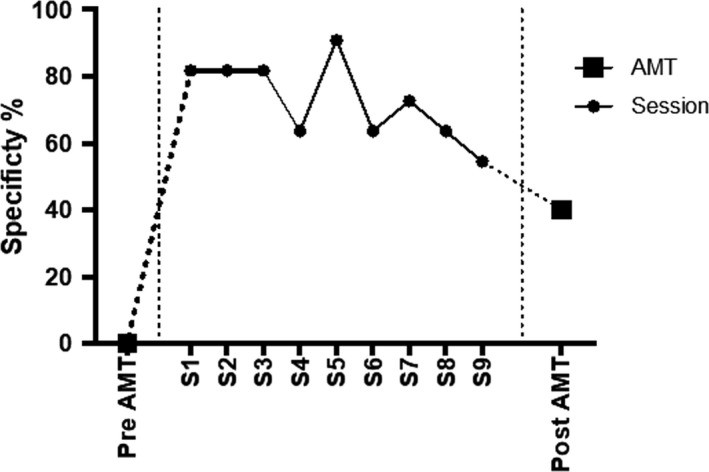
Specificity scores (%) on the Autobiographical Memory Test (pre‐intervention and 1‐month follow‐up measurement) and in‐between session‐to‐session scores on c‐MeST

Figure 2 illustrates the specificity scores of the AMT at pre‐measurement and at 1‐month follow‐up measurement, and specificity scores of c‐MeST sessions in‐between. Specificity scores per session, for neutral, positive, and negative cue words and for exercises of the day can be found in Table 1. Three trends are noticeable. First, although the participant was not able to retrieve specific memories in a face‐to‐face AMT with a time limit, she succeeded at scoring 81.82% already in Session 1 of the computerized training when she was alone, at home, and without time pressure. Second, there was no clear increasing trend from session to session, with scores varying between 54.55% and 90.91% with a mean of 72.73%. Third, of the four types of memory exercises, scores for memories of the day (one for yesterday and one for today) were the highest and a ceiling effect was reached (*M* = 100% across 18 exercises), while scores for negative (*M* = 77.78%) and positive (*M* = 70.37%) cue words were higher than neutral words (*M* = 51.85%).

**Table 1:** Specificity scores for each of the nine sessions of computerized Memory Specificity Training

Session	Total score	Scores for neutral cues	Scores for positive cues	Scores for negative cues	Scores for *memories of the day*
1	9	2	2	3	2
2	9	2	3	2	2
3	9	2	2	3	2
4	7	1	3	1	2
5	10	2	3	3	2
6	7	1	2	2	2
7	8	2	1	3	2
8	7	2	2	1	2
9	6	0	1	3	2
Total	72	14	19	21	18
% Total	72.73%	51.85%	70.37%	77.78%	100%

Note

Each c‐MeST session includes 11 exercises: three exercises of each kind of cue (neutral, positive, and negative values) and two exercises with *memories of the day*.

### 3.3 Symptoms

Comparison of pre‐intervention and follow‐up measurement shows that manic symptom scores on the ASRM‐NL decreased from 10 to 3. A sum score of 6 or higher indicates a high probability of a manic or hypomanic state.

**Figure 3:**
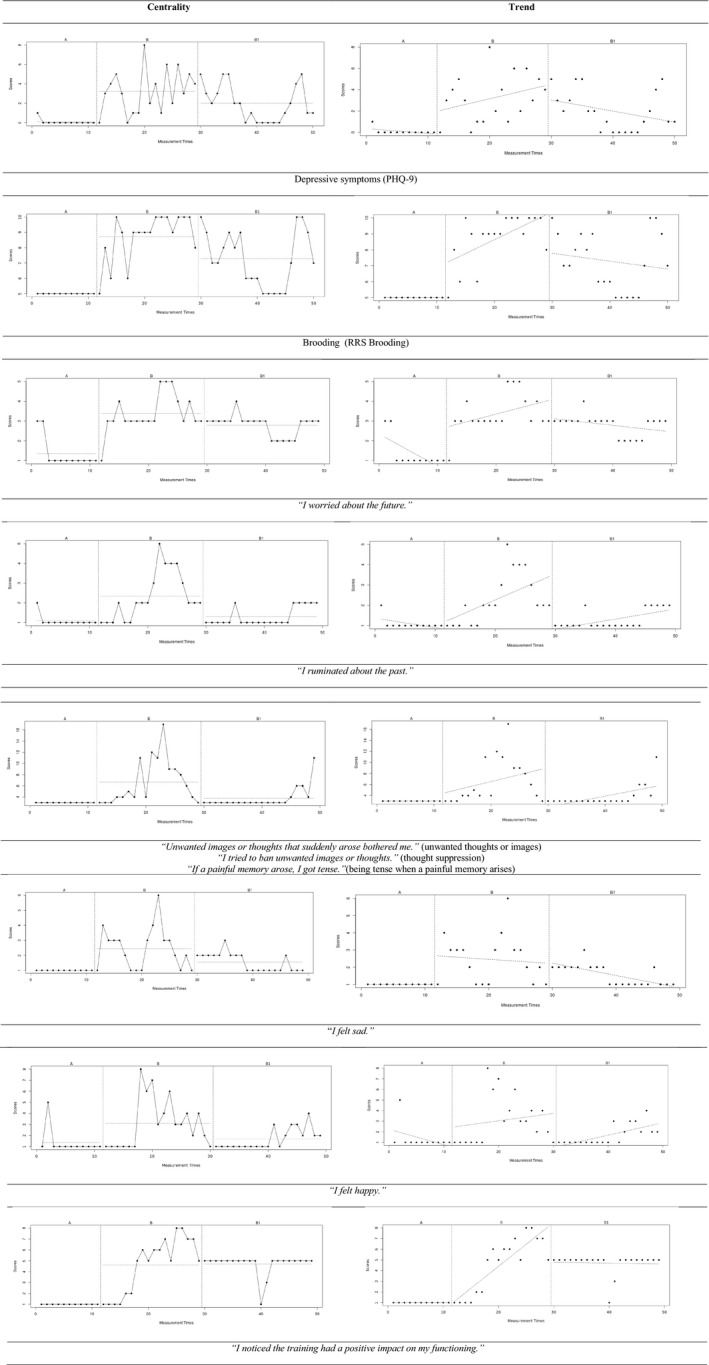
Visual analysis of ABA design showing (1) level of each phase (baseline–training–follow‐up) using mean scores and showing (2) the trend of each phase using least squares regression. For depressive symptoms (PHQ‐9), Brooding (RRS‐5), worrying about the future, ruminating about the past, a combination of the three items inspired by the revised Impact of Events scale (unwanted thoughts or images, thought suppression, being tense when a painful memory arises), sadness, happiness, and experiencing an impact of the training

Visual analysis (Figure 3) of the repeated measures of depressive symptoms (PHQ‐9), sadness, and happiness with the levels of each phase using mean scores and with the trend of each phase using least squares regression suggests three things. First, during baseline the participant reported almost no depressive symptoms, sadness, or happiness. The increased hypomanic state at the beginning of the training might explain why these states were not recognized and not reported by the participant. Second, during the training phase (Phase B, from measurement time 10 to 27) the participant started to report more depressive symptoms, happiness, and sadness. Third, after the training phase during follow‐up, all symptoms show a declining trend again.

### 3.4 Processes

Visual analysis (Figure 3) of all repeated measures of related processes (RRS Brooding, single item on rumination, single item on worrying about the future, the combination of three single items reflecting the IES‐R items) shows the same pattern as in symptoms: These processes were not reported by the participant during baseline, an increase occurred during training phase, and during follow‐up a decline can be observed.

There was no apparent difference in the impact of negative events scores from pre‐intervention (IES‐R = 20) to follow‐up (IES‐R = 22). Both scores are below the cutoff of being of clinical concern. The impact of intrusive prospective (IFES) imagery scores decreased from pre‐intervention (IFES = 31) to follow‐up (IFES = 12). At both time points, the participant provided three positive events (perhaps due to their hypomanic state). Unfortunately, no item of the repeated measures can be regarded as a parallel measure, but the variability on the item concerning worrying about the future suggests that there may have been a similar trend of increase during the repeated measures assessments and then decrease on the follow‐up assessments, as was evident for other study variables.

## 4 DISCUSSION

This case study was the first to examine the potential benefits of remediating rAMS in BD with memory specificity training. rAMS is a transdiagnostic clinically relevant phenomenon, and accumulating evidence has shown the potential of remediating rAMS in other clinical disorders with concomitant effects of disorder symptoms and associated processes (Barry et al., 2019).

For the patient who participated in the present investigation, c‐MeST did improve the skill of retrieving specific memories in a face‐to‐face context with a time limit from pre‐intervention to 1‐month follow‐up measurement. However, results are limited: The participant's score at follow‐up is still in the range of what we would define as rAMS (i.e., poor memory specificity). In addition to the limited effect, the session‐to‐session scores indicate that at intervention Session 1 the participant was able to retrieve specific memories (mean score of 81.82%), and no further improvement was observed throughout the remainder of the training (with scores ranging from 54.55% to 90.91% with a mean of 72.73%). This finding is in line with the results of our other studies using the same training platform with 20 participants with a history of UD (Martens, Barry, Barry, Takano, Onghena, & Raes, 2019) and 20 healthy older adults (Martens, Takano, et al., 2019). This offers no clear information on optimal dose. However, it suggests that the context wherein participants are required to retrieve specific memories could contribute to the ability to do so, such as the presence of another person or a time limit. But even if this is the case, the online version improved the skill sufficiently to have an improved score at 1‐month follow‐up in‐person measurement.

The effects of c‐MeST on repeatedly measured related symptoms and related processes were mixed. The participant barely reported symptoms during baseline, presumably due to hypomania, which is not surprising given her BD type 1 rapid cycling diagnosis. At the start of the training, symptoms then increased (and it is noted that after hypomania symptoms of depression can only worsen by definition). One possible additional interpretation worth considering is in line with the generalized affect regulation strategy hypothesis and the functional avoidance model hypothesis. If rAMS serves the function of affect regulation and of avoidance of thinking of specific memories, MeST could lead to an increase in symptoms in the short run as this avoidance function is challenged. As previous examinations of c‐MeST only included pre‐ and postintervention assessments, it is unclear whether an increase in symptoms during training would be problematic. This discussion is in line with the question on the temporary exacerbation of symptoms in PTSD treatments (Foa, Zoellner, Feeny, Hembree, & Alvarez‐Conrad, 2002). Here however, it is not possible to disentangle what are due to depression symptoms worsening as the participant (who was rapid cycling) came out of hypomania and what may be due to MeST.

The higher specificity scores for emotional cue words versus neutral cues were explained by the participant as due to the difficulty they had in recalling any memories related to the neutral cue words, which they reported as feeling “flat.” Future research can be aimed at finding trends in people with BD, such as the role of valence and/or emotionality of cue words at assessment and during training. For a recent discussion on how cue words of different valences generate different results, see the meta‐analysis and systematic review of MeST by Barry et al. (2019). Alternatively, the c‐MeST software makes it possible to offer more cue words of a certain valence depending on personal scores. A flexible online training tool using an adaptive system might start with cue words chosen by the participant, but after a while when participants' scores increase, the system could motivate the participant to try different cue words of valences for which they have particular difficulty retrieving memories. The present investigation tested a computerized, online, version of MeST, which the participant experienced as feasible: The cue words were judged as not too difficult and the feedback from the classifier was judged as correct. Although the duration of the sessions was experienced as too long, the participant completed out all sessions. For research purposes, we designed the online training such that the participant trained many exercises in a short period of time: 99 exercises in 17 days, as opposed to 104 exercises in 4 weeks in original group version of MeST (Raes, Williams, et al., 2009). In future examinations and implementations of MeST, researchers and clinicians could utilize the functions of the software that permit individualization, where participants and clinicians can choose when and how much to train. In comparison with other in‐person group MeST protocols, this version did not include psycho‐education on memory problems related to psychopathology or psycho‐education and exercises on how to notice when one is thinking in an overgeneral manner and how to interrupt that process (STOP model, session four), nor any therapist‐plus‐group interaction. This might be included as a useful addition in future examinations of c‐MeST or MeST in BD.

The main limitation of this study is that it concerns only one, well‐educated and self‐selected patient. Although some improvement in specificity was observed in the present case study, future research is now needed to examine the impact of MeST or c‐MeST on more diverse groups of people with BD showing rAMS. In the recruitment of participants, we identified 12 patients who recognized the described phenomenon of rAMS and expressed an intention to participate; however, only two patients showed rAMS when tested with the AMT. All other patients with BD were able to retrieve seven or more specific autobiographical memories in a time limited face‐to‐face test. The mean score on AMT (79.17%) is in contrast to previous studies (40.00% (Mowlds et al., 2010); 33.33% (Tzemou & Birchwood, 2007); and 44.5% in remitted BD (Mansell & Lam, 2004)). This discrepancy does not seem to be caused by the use of another form of AMT (all verbal forms including positive and negative cues) or a much smaller sample size (*n* = 12 versus *n* = 52 (Mowlds et al., 2010); *n* = 29 (Tzemou & Birchwood, 2007); *n* = 19 (Mansell & Lam, 2004)). One potential explanation is a sampling bias. Our participants were self‐selected: They recognized the phenomenon rAMS and hoped they could be included in a study examining a training to remediate this process. An increase in motivation at intake might explain an increase in executive function, and thus an increase at retrieving specific memories. Overall, it seems that there is mixed evidence for rAMS in BD, and more research may be needed according to current mood state and type of BD. Also, in future studies it may be useful for someone to come out of an acute (hypomanic, manic, or depressive) episode before taking part in a new intervention.

For this study, standard c‐MeST is used (Martens, Takano, et al., 2019; Takano, Moriya, et al., 2017). One potential interesting route is to adapt it to people with BD by taking into account unique characteristics of memory retrieval in BD (Barry, Chiu, et al., 2018). For example, as discussed previously, vivid mental imagery as part of episodic recall can act as an emotional amplifier of mood states in BD (Holmes et al., 2016). Holmes et al. (2011) also discuss negative mental imagery of the future (“flashforwards”) as a potential important process in BD. Flashforwards, for example, to suicidal acts (Hales, Deeprose, Goodwin, & Holmes, 2011) or future manic events (Ivins, Di, Close, Goodwin, & Holmes, 2014) may also have a past memory component. Whether training rAMS affects mental imagery of the past or future remains to be further examined. For this participant, scores on an ad hoc translated Impact of Future Events Scale decreased from 31 to 12. A potential explanation for this finding is that by instructing participants to retrieve specific memories in a structured and detailed manner, they increased in their ability to manage intrusive thoughts. Deliberate memory responses to cue words (akin to our training here) and experiencing involuntary, intrusive imagery are distinct phenomena according to Holmes, Lang, Moulds, and Steele (2008). Research combining MeST and imagery training has been started in UD (Pile et al., 2018) and might also be of interest in the context of BD.

## 5 CONCLUSIONS

One patient with BD type I rapid cycling and rAMS (a score of 0/10 at the AMT) successfully conducted an online version of Memory Specificity Training (c‐MeST). This led to an increase in memory specificity between pre‐intervention and follow‐up measurement, but also to a deterioration in some repeatedly measured associated symptoms and processes during the training phase. Overall, the participant managed to successfully complete a large number of sessions independently and gave very useful feedback, which is promising in terms of developing a low intensity, process specific psychological intervention approach. Future research should now pilot test this intervention in a group of patients, considering the current stage of the BD cycle that they are in during the study and training phases and the effects of this on their specificity and other associated processes and symptoms. These studies should preferably include patients with more diverse educational backgrounds and BD symptomatology than that of the participant included in this study, as this participant was a highly motivated self‐selected well‐educated patient.

## CONFLICT OF INTEREST

The following facts may be considered as potential conflicts of interest. FR is one of the developers of the original in‐group face‐to‐face MeST. KT, KM, and FR are the developers of the Web‐based computerized MeST (c‐MeST). KM and FR additionally receive payments for training workshops and presentations related to MeST. EH receives book royalties from Guilford Press (for the book Imagery‐Based Cognitive Therapy for Bipolar Disorder and Mood Instability) although this does not include MeST. We wish to confirm that there are no other known conflicts of interest associated with this publication and there has been no significant financial support for this work that could have influenced its outcome.

## AUTHORS' CONTRIBUTIONS

FR is the principal investigator for the study protocol. KM and FR generated the idea and designed the study. KM and SW were responsible for the acquisition of data. KT, TB, and EH made substantial contributions to the analysis and interpretation of data. KM drafted the manuscript. TB, KT, EH, SW, and FR substantively revised it. All authors read and approved the final manuscript.

## Data Availability

All data are included in the manuscript or in appendices.
