# Supplementary material for: Remediating reduced memory specificity in bipolar disorder: A case study using a Computerized Memory Specificity Training
Source: Brain Behav. 2019 Nov 20;9(12):e01468. doi: 10.1002/brb3.1468 (PMC6908894; doi:10.1002/brb3.1468)
Supplement: Supplementary file 1 [file BRB3-9-e01468-s001.docx]

## Measures

Autobiographical Memory Specificity**.** Outside of training sessions, Autobiographical Memory Specificity was measured using a verbal version of the Autobiographical Memory Test with two sets of cues for pre-intervention and one month follow-up assessment (Dalgleish et al., 2007; Williams & Broadbent, 1986). Ten cue words (five positive, five negative, presented in alternating order, presented in Supplementary File 1) were verbally presented. In response to each cue word, the participant was instructed to retrieve a specific memory. It was explained that the memory does not have to be an important event, but needs to be specific, happened once, lasted shorter than a day and happened more than a week ago. Within the instructions, examples of specific and non-specific responses were given and a practice trial with three cue words with feedback took place. The AMT was scored during the verbal interview by the first author, unspecific and unclear answers were followed with prompts until the participant succeeded in retrieving a specific answer or until one minute had passed. The main index of the AMT is the number of cue words for which the participant’s first answer classifies as a specific autobiographical memory, ranging from 0 to 10.

Depressive symptomatology. The Patient Health Questionnaire 9 (Kroenke, Spitzer, Williams, & Löwe, 2010) (Dutch Translation) was used to measure depressive symptomatology. The PHQ-9 is a nine-item self-report measure of depressive symptoms, scoring the nine DSM-5 Major Depressive Episode criteria based on the frequency with which they have been experienced in the last two weeks, from 0 (not at all) to 4 (nearly every day). The PHQ-9 is used as a repeated measurement during the study (baseline, intervention phase and follow up phase), for which the instructions were changed and the participant was asked the frequency which she experienced the symptoms since previous measurement. Scores can vary from 0 to 27.

Rumination*.* The Ruminative Response Scale – Brooding subscale(Raes et al., 2009; Treynor, Gonzalez, & Nolen-Hoeksema, 2003) was used to measure depressive rumination. The RRS Brooding subscale is a self-report questionnaire consisting of five items that are considered to measure maladaptive ruminative thinking including the passively comparing of one’s situation with some unachieved standard. Items ask to report how frequently on a 1 (almost never) to 4 (always) scale someone tends to respond, when in a sad mood, with thoughts like “Why do I always react this way?” or “Why do I have problems other people do not have?”. Scores vary from 5 to 20. The RRS Brooding was used as a part of the repeated measures.

Manic Symptoms. Altman Self-Rating Mania Scale (Altman, Hedeker, Peterson, & Davis, 1997) (ASRM-NL, Dutch translation) is a self-rating mania scale existing out of five multiple-choice items about symptoms compatible with DSM IV criteria of BD, experienced in the past week, with total scores varying from 0 to 20. A sum score of 6 or higher indicates a high probability of a manic or hypomanic condition. ASRM-NL is used as a pre-intervention and follow-up measurement.

Impact of Events*.* Impact of Event Scale – Revised (IES-R; Dutch translation by TZP Psychotrauma 2006)(Weiss & Marmar, 1995) was used as a symptom outcome measure. The IES-R is a 22 item self-report questionnaire measuring symptoms of posttraumatic intrusion (8 items), avoidance (8 items) and hyperarousal (6 items) in the previous week. Items are scored from 0 (not at all) to 4 (a lot), sores vary from 0 to 88. IES-R was used as a pre-intervention and follow-up measurement.

Impact of intrusive prospective imagery*.* Impact of Future Events Scale (Deeprose & Holmes, 2010) (IFES; translated ad hoc to Dutch). IFES provided an index of the impact of intrusive prospective imagery. First, the question was asked to ‘‘Please identify three future events which you have been thinking about by imagining over the past seven days (e.g., positive or stressful life events). For each event, please indicate whether your imagining of it was positive or negative’’. Second, 24 items to assess intrusive pre-experiencing, avoidance, and hyper-arousal were offered and anchored from 0 (not at all) to 4 (extremely). Scores vary from 0 to 96. The IFES was used as a pre-intervention and follow-up measurement.

Items for Repeated Measurements. Eight single items were added to the online platform for repeated measures that capture process change: worrying about the future, worrying about the past, intrusion, avoidance, arousal, sadness and happiness. For this, eight statements were rated on a Likert scale from 1 (*not at all*) to 9 (*almost all of the time*): (a) “I worried about the future”, (b) “I worried about the past”, (c) “Unwanted images or thoughts that suddenly arose bothered me”, (d) “I tried to ban unwanted images or thoughts”, (e) “If a painful memory arose, I got tense”, (f) “I felt sad”, (g) “I felt happy”. Items (c), (d) and (e) mirror the three subscales or IES-R and therefore are combined for analysis.

Measures of c-MeST training experiences. After each c-MeST session the participant was asked three closed and two open questions; (a) to what extent she found the offered words helpful/easy to help retrieve a specific memory (0 = not easy at all, words are very difficult to retrieve memories for, 10 = very easy, words are very easy to retrieve memories for), (b) to what extent has she experienced the feedback of the classifier as correct (0 = not at all, a lot of mistakes, 10 = very correct, no mistakes), (c) to what extent has she experienced the session okay in length (1 = way too short, 2 = a bit too short, 3 = just right, 4 = a bit too long, 5 = way too long), (d) how has she experienced the training and (e) if she had any other remarks.

References

Altman, E., Hedeker, D., Peterson, J., & Davis, J. (1997). The Altman self-rating mania scale. *Society of Biological Psychiatry*, *42*, 948–955.

Dalgleish, T., Williams, J. M. G., Golden, A.-M. J., Perkins, N., Barrett, L. F., Barnard, P. J., … Watkins, E. (2007). Reduced specificity of autobiographical memory and depression: The role of executive control. *Journal of Experimental Psychology: General*, *136*, 23–42. https://doi.org/doi:10.1037/0096-3445.136.1.23

Deeprose, C., & Holmes, E. A. (2010). An exploration of prospective imagery: The impact of future events scale. *Behavioural and Cognitive Psychotherapy*, *38*, 201–209. https://doi.org/10.1017/S1352465809990671

Kroenke, K., Spitzer, R. L., Williams, J. B. W., & Löwe, B. (2010). The Patient Health Questionnaire Somatic, Anxiety, and Depressive Symptom Scales: A systematic review. *General Hospital Psychiatry*, *32*, 345–359. https://doi.org/doi:10.1016/j.genhosppsych.2010.03.006

Raes, F., Schoofs, H., Hoes, D., Hermans, D., Van Den Eede, F., & Frank, E. (2009). ‘Reflection’ en ‘brooding’ als subtypes van rumineren: een herziening van de Ruminative Response Scale. *Gedragstherapie*, *42*, 205–214.

Treynor, W., Gonzalez, R., & Nolen-Hoeksema, S. (2003). Rumination reconsidered: A psychometric analysis. *Cognitive Therapy and Research*, *27*, 247–259. https://doi.org/doi:10.1023/A:1023910315561

Weiss, D. S., & Marmar, C. R. (1995). Impact of Events Scale - Revised. *Assessing Psychcological Trauma and PTSD: A Practitioner’s Handbook N.Y: Guildford, 1995.*

Williams, J. M. G., & Broadbent, K. (1986). Autobiographical memory in suicide attempters. *Journal of Abnormal Psychology*, *95*, 144–149. https://doi.org/doi:10.1037/0021-843X.95.2.144
