# Supplementary material for: Remediating reduced memory specificity in bipolar disorder: A case study using a Computerized Memory Specificity Training
Source: Brain Behav. 2019 Nov 20;9(12):e01468. doi: 10.1002/brb3.1468 (PMC6908894; doi:10.1002/brb3.1468)
Supplement: Supplementary file 2 [file BRB3-9-e01468-s002.docx]

*Results for three feasibility questions; (a) “have you found the offered words today easy to help you retrieve a specific memory?” (0 = not easy at all, very difficult words, 10 = very easy, easy words), (b) “have you experienced the feedback as correct?” (0 = not at all, a lot of mistakes, 10 = very correct, no mistakes), and (c) “have you experienced the session of today okay in length?” (1 = way too short, 2 = a bit too short, 3 = just right, 4 = a bit too long, 5 = way too long).*

|  | Q1: Difficulty of Cue Words | Q2: Correctness Classifier | Q3: Length of Session |
| --- | --- | --- | --- |
| Set 1 | 5 | 7 | 4 |
| Set 2 | / | / | / |
| Set 3 | 3 | 9 | 4 |
| Set 4 | 7 | 10 | 3 |
| Set 5 | 7 | 8 | 3 |
| Set 6 | 7 | 9 | 4 |
| Set 7 | 8 | 9 | 4 |
| Set 8 | 3 | 9 | 3 |
| Set 9 | 4 | 9 | 4 |
| ***M* (SD)** | **5.5 (2.00)** | **8.75 (.89)** | **3.63 (.52)** |
